# Life and death of a single catalytic cracking particle

**DOI:** 10.1126/sciadv.1400199

**Published:** 2015-04-03

**Authors:** Florian Meirer, Sam Kalirai, Darius Morris, Santosh Soparawalla, Yijin Liu, Gerbrand Mesu, Joy C. Andrews, Bert M. Weckhuysen

**Affiliations:** 1 Debye Institute for Nanomaterials Science, Faculty of Science, Utrecht University, Universiteitsweg 99, 3584 CG Utrecht, Netherlands.; 2 Stanford Synchrotron Radiation Lightsource, SLAC National Accelerator Laboratory, 2575 Sand Hill Road, Menlo Park, CA 94025, USA.; 3 Research Centre for Catalysts, Albemarle Corporation, 13000 Baypark Road, Pasadena, TX 77507, USA.

**Keywords:** Chemistry, Catalysis, Catalyst deactivation, Fluid Catalytic Cracking, Crude Oil Processing, X-ray Microscopy, Chemical Imaging, Metal Poisons, Zeolite

## Abstract

Fluid catalytic cracking (FCC) particles account for 40 to 45% of worldwide gasoline production. The hierarchical complex particle pore structure allows access of long-chain feedstock molecules into active catalyst domains where they are cracked into smaller, more valuable hydrocarbon products (for example, gasoline). In this process, metal deposition and intrusion is a major cause for irreversible catalyst deactivation and shifts in product distribution. We used x-ray nanotomography of industrial FCC particles at differing degrees of deactivation to quantify changes in single-particle macroporosity and pore connectivity, correlated to iron and nickel deposition. Our study reveals that these metals are incorporated almost exclusively in near-surface regions, severely limiting macropore accessibility as metal concentrations increase. Because macropore channels are “highways” of the pore network, blocking them prevents feedstock molecules from reaching the catalytically active domains. Consequently, metal deposition reduces conversion with time on stream because the internal pore volume, although itself unobstructed, becomes largely inaccessible.

## Teaser

Macropore blocking through metal deposition and intrusion of particles is a major deactivation mechanism in FCC catalysts essential to gasoline production.

## INTRODUCTION

Catalytic cracking is an essential process for gasoline production and for the manufacturing of base chemicals, such as propylene. Currently, more than 450 fluid catalytic cracking (FCC) plants are operated worldwide (*1*), consuming about 2000 metric tons of catalyst per day (*2*). In FCC, long-chain feedstock molecules are cracked into smaller, more valuable ones by hierarchically structured multicomponent catalyst particles consisting of zeolite, matrix, filler, and binder (*3*–*5*). Microporous crystalline zeolites provide most of the catalytic activity and product selectivity. They are embedded in the particle matrix, which itself plays an active role in precracking large hydrocarbons to appropriate size so that they can enter zeolite micropores for further selective cracking. The filler consists of clay used to dilute catalyst particle activity, whereas the binder holds together zeolite, matrix, and filler.

During commercial FCC, the particles accumulate metals from crude oil and/or upstream processes and equipment. Ni and V contaminants are predominantly present in conventional heavy crude oils, whereas Fe contaminants are known to be present at relative high concentrations in tight and/or shale oils. Metal accumulation occurs via deposition and incorporation into the catalyst body and can cause unwanted shifts in product distribution, destabilize zeolite domains (*6*–*8*), and reduce bulk accessibility (*9*, *10*). We summarize these effects as “catalyst aging.” Demetallization methods to mitigate metal deposition have been proposed (*11*); however, to successfully commercialize such techniques, it is crucial to understand metal behavior throughout the FCC particles. Methods studying particles in bulk provide averaged metrics on properties such as metal content, acidity, porosity, and pore accessibility (*6*, *12*, *13*). At the single-particle level, our previous work (*14*–*17*) has focused on correlating structural damage to loss of active acid sites within single FCC particles. Imaging techniques, such as scanning electron microscopy–energy-dispersive x-ray spectroscopy (SEM-EDX) (*10*, *18*, *19*), have been used to study metal distribution along selected portions of particle surfaces. However, until now, a study attempting to understand metal pore intrusion and its suggested relation to macro- and mesopore blockage has remained elusive because it requires not only a tomographic technique but also the capability to image whole particles to assess pore connectivity. Electron microscopic (EM) tomography has been conducted on zeolites (*20*), but EM is not sufficiently penetrative to study whole FCC particles with diameters of 40 to 150 μm. Micro–computed tomography (CT) has provided morphological information for multiple FCC particles at micrometer spatial resolution, whereas nanotomography was applied only to a subportion of a single particle (*21*, *22*). To study macroporosity [that is, pore sizes down to ~50-nm diameter (*23*)] and pore connectivity, a nondestructive method with sufficient spatial resolution, a field of view (FOV) of several tens of square micrometers, and the capability to simultaneously map three-dimensional (3D) metal distributions is required to elucidate the suggested connection between metal deposition and changing macroporosity. On the basis of the advances in x-ray microscopy in the last decade, synchrotron-based hard x-ray nanotomography today approaches the required spatial resolution and meets the other criteria for imaging catalytic solids (*21*, *22*, *24*–*31*).

## RESULTS

### Catalyst aging inspected at the bulk and single-particle level

**Fig. 1:**
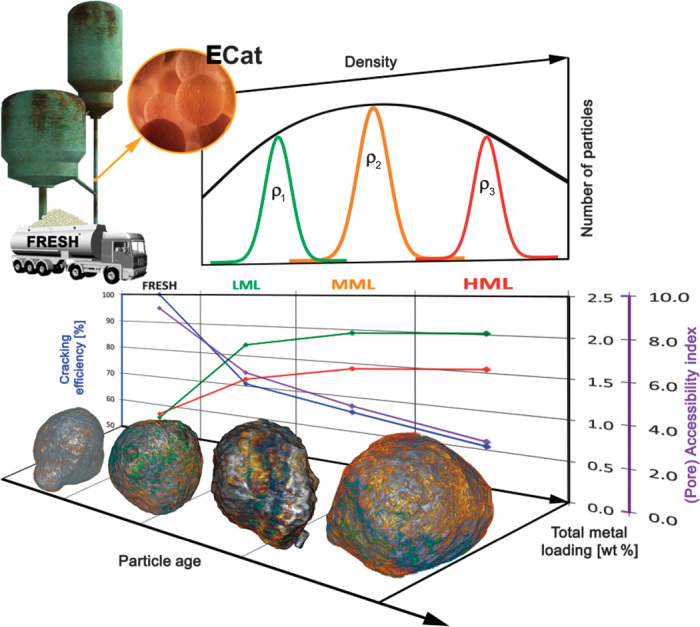
Bulk analysis results of FRESH, LML, MML, and HML FCC catalyst particles. (**Top**) Fresh catalyst particles and calcined ECat particles were obtained from an industrial FCC unit and separated into three age groups according to their skeletal density (ρ1 to ρ3). (**Middle**) Catalytic cracking activity (%) (blue) of vacuum gas oil (VGO) and accessibility index (purple) are anticorrelated with the total metal content of Fe and Ni (summed Fe and Ni concentrations in wt %) for particle surface (green) and bulk (red), as measured by SEM-EDX and WDXRF (Table 1). (**Bottom**) Catalytic particles are imaged by TXM tomography at 64-nm 3D voxel size (see the Supplementary Materials). Red to orange colors indicate Fe concentrations, whereas Ni is visualized using the blue to green color range (see also movie S1).

Here, we present the first study of its kind, in which we developed an analytical approach that allowed us to quantify and directly visualize in 3D how Fe and Ni deposition affects particle macropore accessibility at the single-particle level and as a function of increasing metal loading. The latter was achieved by studying fresh FCC particles together with a set of calcined equilibrium catalyst (ECat) particles taken from a commercial FCC unit and subsequently separated into three catalytic age groups (Fig. 1).

The obtained ECat particles were separated on the basis of their skeletal density, making use of the fact that ECat particles from an industrial source show a spread in density distribution (Fig. 1, top). This in turn is related to the fact that the catalyst mixture in an industrial FCC unit has a broad age distribution because of continuous replenishment of fresh catalyst into, and withdrawal of spent catalyst from, the FCC unit. On average, older ECat particles have accumulated more metals from the feed than younger ECat particles because of the longer time spent in the FCC unit, leading to a higher skeletal density of older ECat particles in comparison to younger ones (*32*). However, for studying catalyst aging, the actual particle age is less relevant; it is the catalytic age, that is, the metal loading or degree of poisoning in relation to a decreased catalytic activity, which is the topic of this study.

Grouping catalytic cracking particles according to their catalytic age was realized using a sink-float density separation method and using this correlation of particle skeletal density with metal loading. In the following, each age group (fresh, low, medium, and high metal loading, further denoted as FRESH, LML, MML, and HML, respectively) was characterized using bulk techniques (see Materials and Methods), which revealed an anticorrelation of total metal content with pore accessibility, catalytic activity, micro- and mesopore surface area, and micropore volume (Fig. 1 and Table 1).

**Table 1:** Results of bulk (multiple particle) analysis performed with the density-separated particle fractions. Uncertainties are reported as 1σ and have been estimated from repeated control measurements where possible [unknown SDs are indicated by N/K (not known)]. Nickel and vanadium were not detected (N/D) in SEM-EDX and WDXRF analyses of the FRESH sample. As explained in the text, the conversion of the FRESH sample is assumed to approach 100 wt %.

**Metric**	**FRESH**	**LML**	**MML**	**HML**	**1σ**
Skeletal density (g/cm^3^)	2.785	2.946	2.953	2.957	N/K
SEM-EDX, La (wt %)	1.64	1.70	1.63	1.84	N/K
SEM-EDX, Fe (wt %)	0.27	1.34	1.60	1.61	N/K
SEM-EDX, Ni (wt %)	N/D	0.34	0.35	0.40	N/K
SEM-EDX, V (wt %)	N/D	0.20	0.22	0.29	N/K
WDXRF, La_2_O_3_ (wt %)	2.86	2.81	2.76	2.74	0.02
WDXRF, Fe_2_O_3_ (wt %)	0.34	0.79	0.93	0.96	0.01
WDXRF, NiO (wt %)	N/D	0.33	0.49	0.59	0.01
WDXRF, V_2_O_5_ (wt %)	N/D	0.53	0.66	0.74	0.01
BET surface area (m^2^/g)	262.0	133.9	108.1	93.3	2.62
Micropore surface area (m^2^/g)	131.5	81.6	73.0	62.5	3.66
Micropore volume (cm^3^/g)	0.0605	0.0243	0.0163	0.0140	N/K
Mesopore surface area (m^2^/g)	139.0	52.3	35.1	30.8	3.73
(Pore) accessibility (AAI)	9	4.9	3.5	2.4	0.90
430°F+ conversion (wt %) [CTO: 3 (w/w)]	~100	65.12	57.59	54.52	0.57
430°F+ conversion (wt %) [CTO: 4 (w/w)]	~100	68.16	61.29	58.2	0.57
430°F+ conversion (wt %) [(CTO: 5 (w/w)]	~100	70.91	65.64	60.93	0.57
430°F+ conversion (wt %) [CTO: 6 (w/w)]	~100	74.44	67.46	64.21	0.57

These data confirm the known detrimental effects of metal incorporation and strongly suggest micro- and mesopore clogging by some or all three of the metals Fe, Ni, and V. More specifically, the values reported in Table 1 show that the most drastic changes in sample metrics are consistently observed between the FRESH and the LML group, indicating strong metal accumulation already at an early stage of the catalytic life of FCC catalyst particles. It is striking that the increase in surface and bulk elemental concentration of Fe, as well as the bulk elemental concentration of Ni, is stronger between the LML and MML samples than between the MML and HML samples. This suggests that with increasing total metal loading of those metals, less and less metals are incorporated, possibly approaching a limit of total metal accumulation, that is, approaching a steady state between metal deposition and removal (for example, through surface abrasion). A comparison of results obtained by SEM-EDX and wavelength dispersive x-ray fluorescence spectroscopy (WDXRF) shows that the Fe surface metal concentration becomes dominant with the LML sample (that is, the surface concentration becomes greater than the average bulk sample concentration). In the case of Ni, this does not happen, suggesting that Ni deposition follows different dynamics than the accumulation of Fe.

**Fig. 2:**
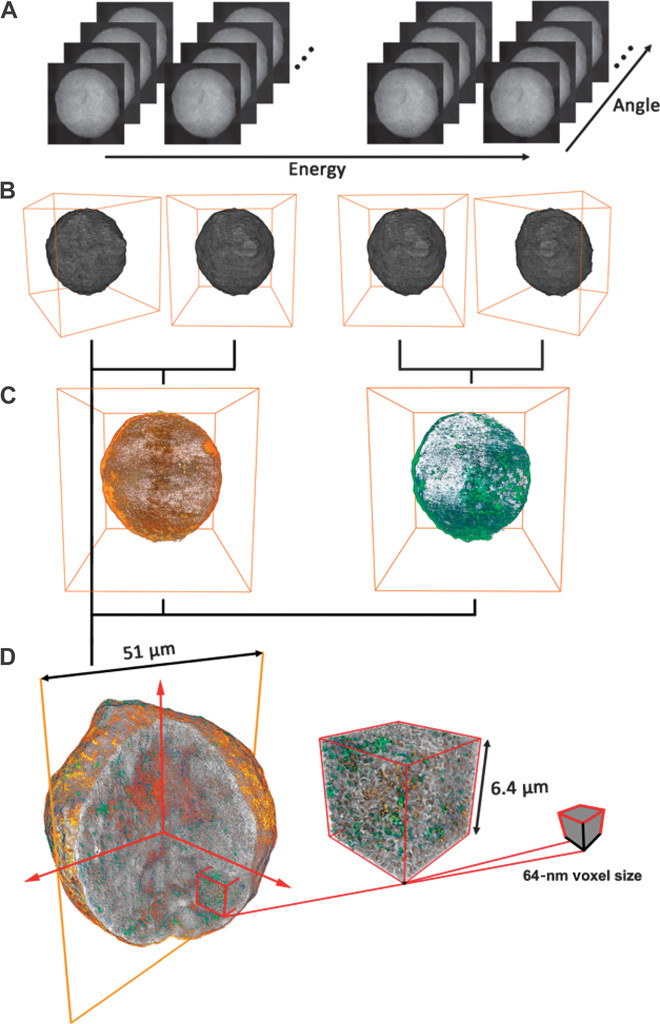
Transmission x-ray nanotomography of FCC catalyst particles. (**A** and **B**) Data are collected below and above the x-ray absorption Fe and Ni K-edge, respectively (A), and reconstructed separately, resulting in four sample volumes (B). (**C** and **D**) Pairwise subtraction of the volumes (differential absorption contrast imaging) provides the 3D distribution of Fe (red) and Ni (green) (C), which is then correlated with pore distribution analysis to reveal the degree of pore clogging by metals and the effects on pore connectivity and accessibility (D). Massive data sets collected and analyzed for every single FCC particle contained, depending on the individual particle size, between 301 and 836 million voxels of 64 × 64 × 64 nm^3^. See movie S1 displaying a fully reconstructed particle illustrating Fe and Ni distributions.

To assess whether similar pore blocking also occurs at the macropore scale, which is difficult to probe for minute bulk sample amounts, we then used mosaic tomography with synchrotron-based full-field transmission x-ray microscopy (TXM) in differential absorption contrast mode (*33*) to image whole, individual FCC particles of each age group at 64-nm 3D voxel size (that is, a cubic volume unit with 64-nm edge lengths), whereas the 3D resolution was estimated to be 314 nm by Fourier ring correlation (FRC; see the Supplementary Materials). One tomography was performed at four distinct x-ray energies to obtain 3D macroporosity as well as Fe and Ni distributions for each individual particle (Fig. 2).

With these massive data sets (between 301 and 836 million voxels at each energy, depending on the catalyst particle size), we were able to (i) inspect single, whole particles of each catalytic age group, (ii) exclusively probe macroporosity, and (iii) individually track changes in macroporosity caused by each metal. The latter is possible because the x-ray absorption contrast for each metal can be minimized (“switched off”) by imaging closely below the x-ray absorption edge of the metal, and maximized (“switched on”) by collecting data slightly above the x-ray absorption edge. The absorption contrast difference between the two data sets therefore directly provides the 3D elemental concentration distribution of the specific metal (*33*). Furthermore, because Fe and Ni are present at low concentrations [on average below 2 weight percent (wt %), Table 1], and because the absorption contrast is minimized below the absorption edge, these metals do not contribute significantly to the absorption contrast recorded at this energy. Therefore, the data recorded below the absorption edge largely show the sample without the metal in question (the metal is switched off). It should be noted that imaging below the Ni x-ray absorption edge means imaging above the Fe edge. Consequently, Fe is switched on and appears as part of the particle matrix when examining Ni (see the Supplementary Materials for more details).

### 3D distribution of Fe and Ni as a function of the catalytic age

**Fig. 3:**
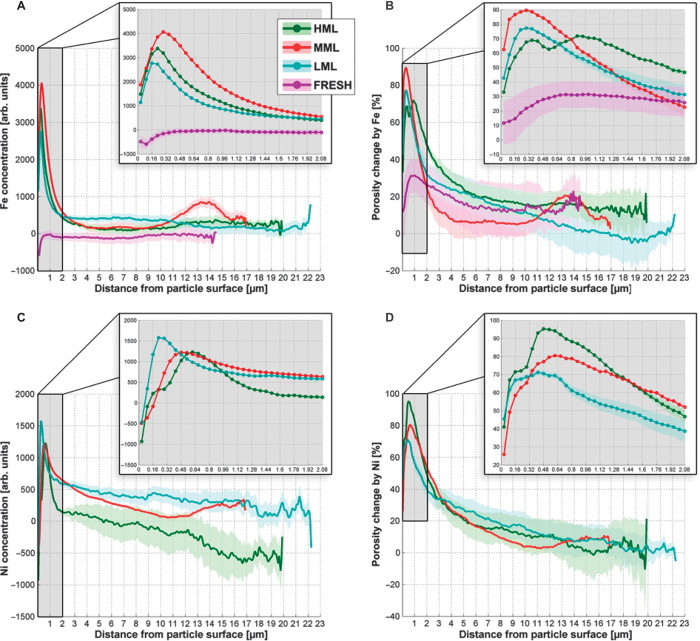
Radial evaluation of single FCC particles. (**A** to **D**) Elemental concentrations of Fe (A) and Ni (C) and porosity changes caused by Fe (B) and Ni (D) in FCC particles of different ages (HML, MML, LML, and FRESH), plotted as a function of distance from the particle surface. For the FRESH particle, only Fe was analyzed because it contained no Ni. Data analysis methods and evaluation of uncertainty used to generate fan plots are reported in the Supplementary Materials.

Calculated single-particle metrics based on sample morphology are listed in table S1. To assess the effect of Fe and Ni on the macropore volume, we report three macropore volumes for each particle: the macropore volume without considering the metal, and one volume each considering the presence of Fe and Ni, respectively. In contrast to the changes seen for microporosity, the changes in macropore volumes and surface areas due to Fe and Ni are very similar not only for both elements but also for all FCC particle ages (within uncertainty levels). This concludes that any possible Fe- and Ni-related changes in macroporosity are too small to be detected when averaged over the whole particle (in the Supplementary Materials, we included a more detailed discussion of this effect, which is method-inherent for highly localized metal concentrations). On the basis of these results and the fact that metal concentrations were reported to show an abrupt decrease within the first few micrometers from the surface (*10*, *18*), we decided to inspect both metal concentrations and macroporosity changes as a function of distance from the particle surface. We calculated these values as the average for all voxels with identical distance from the particle surface, taking into account the actual, individual 3D particle shape. Typically, FCC particles have a denser crust and more porous body [for example, *10*, *18*)], causing porosity to change as a function of depth into the particle. We therefore expressed porosity changes as percent of the porosity measured without considering the specific metal (that is, with the metal switched off). The results of this evaluation are reported in Fig. 3 and confirm that within all ECat particles, Fe and Ni concentrations show a drastic drop within the first 2 μm. The positive correlation between macroporosity change and metal concentration clearly demonstrates that metal deposition is indeed blocking macropores. Moreover, our results show that clogging and deposition happen almost exclusively in the surface layer. Whereas Fe concentrations are generally greatest closer to the surface (Fig. 3A), Ni concentrations peak slightly deeper in the particle (Fig. 3C), particularly for MML and HML. With increasing catalytic age, the Ni plot develops a shoulder that is aligned with the maximum of the Fe concentration. This observation points toward the formation of a ~400-nm-thick Fe-rich layer at the surface of the particles, which alters the incorporation mechanisms for Ni in this region. This is in agreement with the macroporosity change caused by Ni (Fig. 3D), where the plot peaks at ~550 nm, exactly where Ni concentrations (Fig. 3C) reach a maximum and the Fe-caused porosity changes in the HML sample show a local minimum (Fig. 3B). Conversely, a smaller porosity change due to Ni (Fig. 3D) is observed in the Fe-rich surface layer (<400 nm), where the porosity change is predominantly due to Fe (Fig. 3B). These observations indicate the interesting phenomenon that at later stages of the particles’ catalytic life, Fe and Ni are anticorrelated in terms of concentration and macropore clogging.

**Fig. 4:**
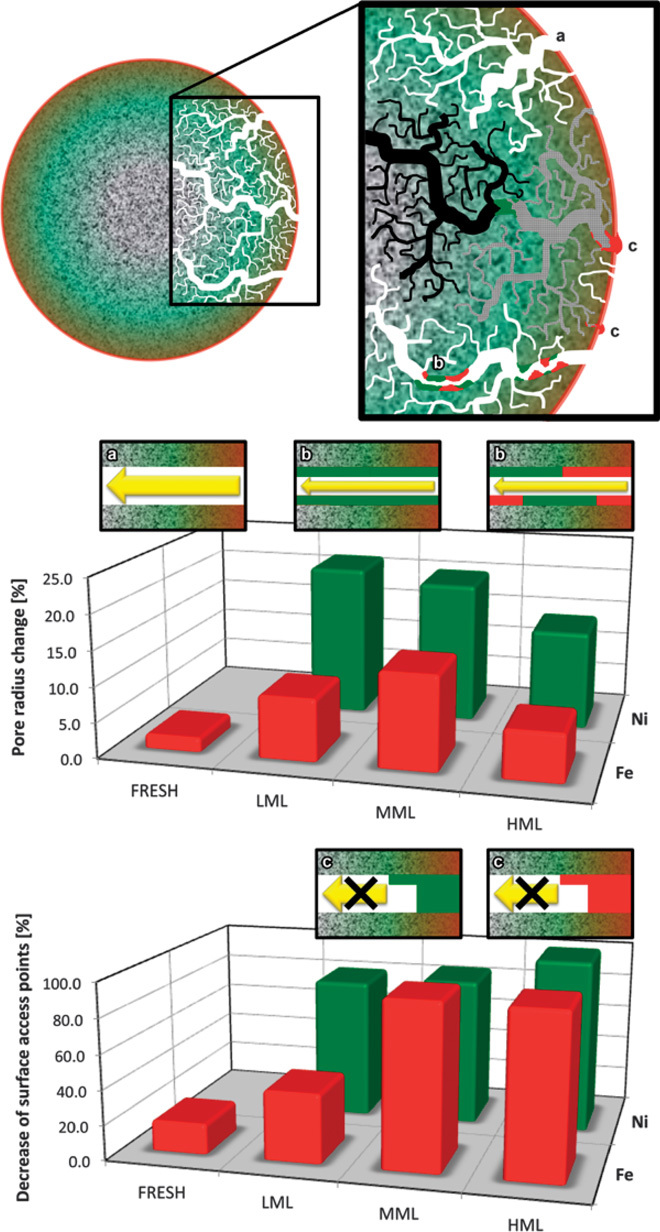
Changes in the pore accessibility of a single FCC catalyst particle as a function of metal content and type. (**Top**) Scheme for metal deposition in the pore network, indicating Fe (red) mainly at the outer surface and Ni (green) penetrating more deeply, as demonstrated in Fig. 3. Pore channels can remain open (a), become narrowed (b), or have blocked accessibility (c) by metal deposition in surface pore access sites. Case “c” may also be that macropores (>64 nm) have been converted to mesopores (<64 nm) that are no longer visible with this method. (**Middle**) Pore radius change in percent as a function of particle age for Fe (red) and Ni (green), respectively. For the FRESH sample, the pore radius change is negligible, indicating that the pore network is not affected by macropore narrowing (that is, case “a” represented by the corresponding inset). The two other insets schematically display how pore narrowing becomes significant for the LML and MML samples and is dominated by Ni in the early phase (LML sample). (**Bottom**) Decrease of surface access points in percent as a function of particle age for Fe (red) and Ni (green), respectively. The two insets schematically depict how at an earlier stage (LML) Ni dominates the reduction of surface macropores that provide access to the internal pore network of the particle (case c), while later (MML and HML), both metals cause severe access restriction through macroporosity.

This severe macropore clogging in the surface layer can have major implications on the accessibility of the catalytically active domains deeper in the particle, because the surface exhibits the entry point for the feedstock. Thus, we investigated related changes in accessibility of the particles’ total macropore volume (TPV). Figure 4 (top) schematically depicts an FCC particle cross section, illustrating the suggested manner of pore filling by Fe (red) and Ni (green) via narrowing (Fig. 4, case b) and blocking of macropore channels in the particle or in the surface layer (Fig. 4, case c). Studies on diffusion within FCC particles have determined that diffusivity increases with pore size; larger pore sizes, particularly macropores, therefore result in higher catalytic activity (*34*, *35*). A decrease in macropore channel radius and number of surface access sites will significantly affect catalyst activity, even when macropores within the largest, central part of the particle remain open.

### Reduced accessibility through metal deposition

To study and quantify such effects, we represented each measured pore space by its topological skeleton (see the Supplementary Materials for analytical details) identified as “pore network” in the following. Table S2 lists some basic parameters of the established pore networks, showing that a single, very large interconnected subnetwork always dominates the macropore network. Remarkably, we found that in all inspected particles, more than 80% of all macropore channels are members of this main subnetwork, which contains more than 90% of the total covered macropore volume. This means that >90% of the macropore space in FCC particles is interconnected, independent of their catalytic age. It becomes clear that an understanding of the relationship between particle macroporosity and activity requires a study of how macropore space is interconnected and how accessible it is from the surface. The reason is that any metal deposition that narrows or completely blocks the macropore channels can cut off previously interconnected subvolumes, making them and the catalytically active domains therein largely inaccessible for feedstock molecules (see movie S2). (Note that access through meso- and micropores might still be possible.)

In the first step of the pore network analysis, we therefore determined metal-caused changes of pore channel radii, thus individually quantifying overall pore narrowing by Fe and Ni (Fig. 4, middle). Considering that metal deposition mainly affects the surface region, the average percent decrease in pore radius due to deposits of Fe and Ni becomes significant for the LML and MML particles. Ni has a stronger effect than Fe, especially for the LML particle (see Discussion).

In the second step, we quantified surface blocking using the number of possible “access points” at the surface of the particles (that is, macropores in the crust of the particle connected to the pore space within). The bottom part of Fig. 4 shows the metal-induced reduction of access points connected to the main subnetwork (which represents >90% of the covered macropore space). The results for both Fe and Ni are in excellent agreement with the trend seen in SEM-EDX data (Table 1): Fe causes a smaller access point reduction in the LML sample, whereas MML and HML show a severe and very similar reduction. Ni always severely blocks access with similar values for LML and MML and a slightly larger value for HML. In the case of HML, both Ni and Fe cause (almost) complete blocking of access (100 and 97.5% access point reductions, respectively; see Table 2). Ni (unlike Fe) continues to accumulate (Table 1) even when many of the surface macropores are blocked. These observations point toward differences in the deposition mechanisms not only between Fe and Ni but also at different stages of the catalytic life of the particles (see Discussion).

**Table 2:** Changes in the single-particle macropore network caused by the metals Fe and Ni. Uncertainties have been determined as the SD of the changes (in percent) calculated using methods 1 and 2 for pore volume determination (see the Supplementary Materials). Nickel was not detected in the FRESH sample; therefore data on changes caused by Ni are not available (N/A).

**Pore volume**	**Metric**	**FRESH**	**LML**	**MML**	**HML**
Fe	Change in average pore radius (%)	2.0 ± 0.9	8.8 ± 4.0	13.3 ± 4.6	6.8 ± 2.6
	Decrease in number of surface access points of largest subnetwork (%)	17.2 ± 9.4	39.3 ± 6.7	94.5 ± 1.6	93.7 ± 0.6
Ni	Change in average pore radius (%)	N/A	21.5 ± 10.5	20.0 ± 2.1	13.9 ± 3.4
	Decrease in number of surface access points of largest subnetwork (%)	N/A	80.9 ± 3.1	84.9 ± 0.9	100.0 ± 0.0

### Particle surface maps

In the first two steps, we used the single, main pore network of each particle to successfully (and for the first time) establish a relationship between Fe and Ni loading (and therefore catalyst age) and changes of the macropore structure in individual FCC particles. In the last step, we extended our analysis to the full particle pore network including all (not interconnected) subnetworks and inspected the effects of Fe and Ni on pore network interconnectivity and accessibility from the surface.

**Fig. 5:**
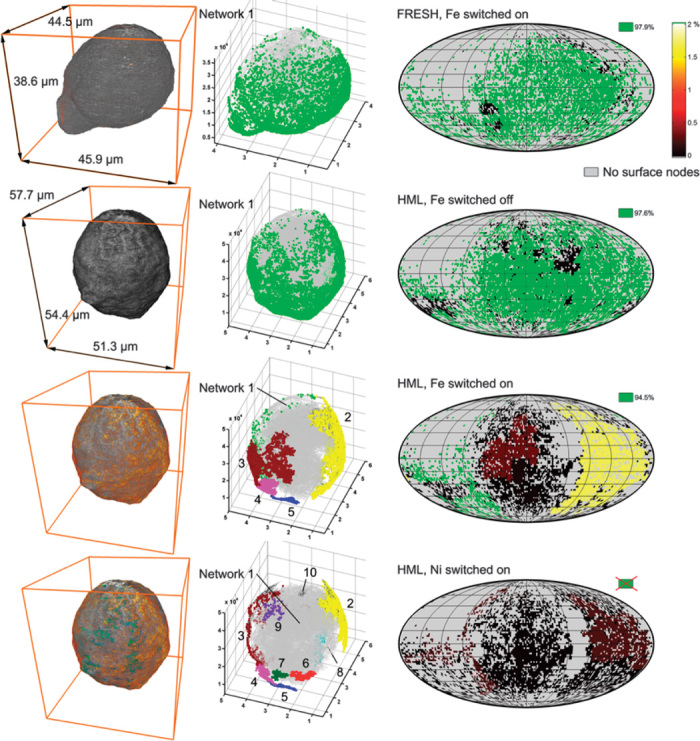
Effects of Fe and Ni on pore network interconnectivity and accessibility from the surface. (**Left**) Rendering of TXM tomography data collected for the FRESH sample including 3D Fe concentrations (top panel) and the HML sample without metals (second panel), including Fe distribution (third panel, same dimensions), and both Fe and Ni (bottom panel, same dimensions). (**Middle**) 3D representation of the established pore networks; for clarity, only the main network is plotted for the FRESH and HML samples in the top and second panel, and the largest 5 and 10 pore networks in terms of volume are plotted in panels 3 and 4 from the top, respectively. (**Right**) Mollweide projections of the particle surface depicting all detected access sites connected to different macropore networks in the particle. Green markers provide access to the largest interconnected macropore subvolume (given in percent of the TPV). For the HML particle, Ni completely blocks surface access to the largest macropore subvolume. Access to smaller macropore subvolumes is indicated by the color scale ranging from 0 to 2% TPV. Gray pixels (0%) refer to areas without surface access points, that is, without detectable macropores. See the Supplementary Materials for analytical details and Fig. 6 for similar maps of correlated penetration depths.

We therefore determined the covered pore volume and the largest accessible distance from the particle surface (in the following called “penetration depth”) for each subnetwork of the macropore network. The accessible pore volume (in percent of the TPV covered by the pore network) was determined as the pore volume covered by each subnetwork connected to the outside of the particle through surface access points. The smallest distance to the particle surface was calculated for all points of the subnetwork, and the maximum penetration depth (in percent of the equivalent spherical diameter of the particle, see table S1) was then determined as the largest of those distances. Then, all surface access sites of each subnetwork were assigned to the pore volume and penetration depth of their corresponding subnetwork. This is, for example, shown in the top row of Fig. 5 for the largest subnetwork in the FRESH sample: each of the light green markers in the central panel represents a surface node (access site) of the main pore network, whereas gray dots indicate nodes of the same network that are located deeper within the particle. Hence, these (gray) nodes are accessible from the outside of the particle through the (green) surface access sites.

**Fig. 6:**
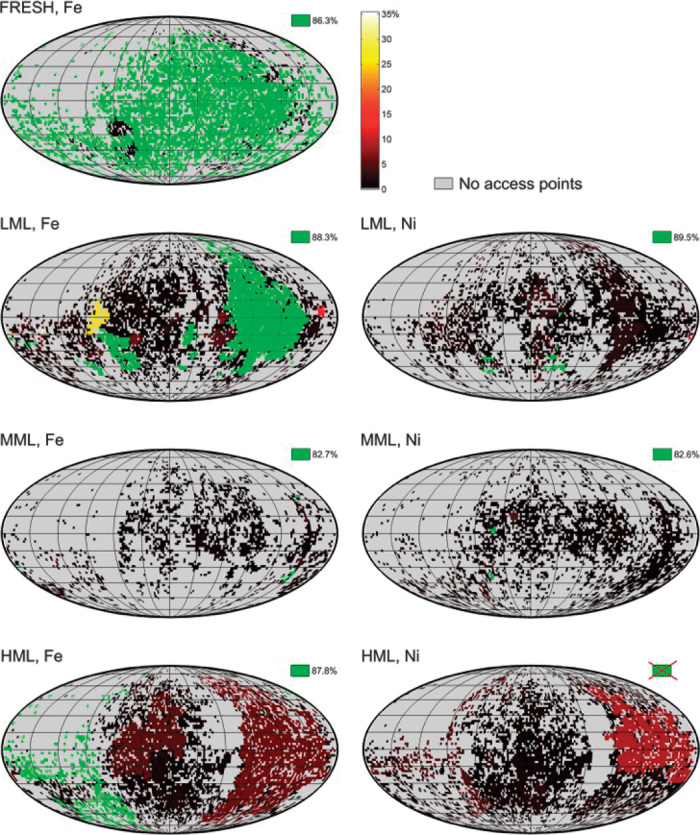
2D surface maps visualizing access points to the macropore volume of single FCC catalyst particles—penetration depth. Effect of metal poisoning by Fe (**left**) and Ni (**right**) restricting access to the FCC particle pore volume, visualized as a 2D surface map (Mollweide projection). Dots indicate remaining access sites after considering the access-blocking effect of the metals. Green markers provide access to the largest interconnected macropore subvolume and therefore allow the entering feedstock molecules to reach the largest penetration depth (given in percent of the particles’ equivalent spherical radii, see table S1). For the HML particle, Ni completely blocks surface access to the largest macropore subvolume. The color scale ranging from 0 to 35% of the equivalent spherical particle radius indicates access to smaller macropore subvolumes. Gray pixels (0%) refer to areas without surface access points, that is, without detectable macropores.

Finally, all surface access sites were mapped onto a 2D image using a Mollweide projection. In these particle surface maps, each surface access point is assigned a color representing the corresponding values of accessible pore volume (Fig. 5) or penetration depth (Fig. 6) established for the subnetwork to which the surface access site connects. This generates a map of the particle surface showing possible entry points for feedstock molecules and the size of the pore volume they can access (Fig. 5) or how deep they can penetrate into the particle (Fig. 6). Because the values of the largest subnetwork are always much larger than those of all other subnetworks, they are assigned the color green, whereas all other surface access points are assigned a color ranging from black to red and yellow to white covering values up to 2% (Fig. 5) or 35% (Fig. 6). Areas where no access sites exist are indicated in gray. The latter areas therefore indicate surface areas where the feedstock cannot enter the particle volume through macropores.

In Fig. 5 where we compare data for the FRESH and HML samples, it is striking that the pore network of the HML sample, without considering the pore clogging effects of Fe and Ni (both switched off), resembles the pore network of the FRESH sample, even though Fe is switched on there. When Fe is switched on in the HML sample, the situation changes drastically: most access points that were part of the main pore network (network 1, light green markers) become disconnected and are now part of much smaller subnetworks, each accounting for less than 2% of the total covered macropore network (as indicated by the color scale bar). Some access points are even completely removed by Fe deposition (gray surface areas). As already shown in Fig. 4, the situation is even more dramatic when considering the effect of Ni, which reduces the number of access points to the main pore network (network 1, see Fig. 5) to zero. As depicted in the last row of Fig. 5, most surface access sites still exist, but because they are now disconnected from the large, inner macropore network of the particle, they only provide access to less than a few percent of the macropore space in the FCC particle. In Fig. 6, the effect of metal deposition is visualized for all age groups, showing how accessibility (in this case, quantified using the penetration depth) decreases with increasing catalyst particle age. For particles with higher metal loading, only few access sites (green markers) remain, which directly visualizes how macropore access for feedstock molecules becomes severely limited by metal poisoning.

## DISCUSSION

Our results are in excellent agreement with earlier 2D studies of FCC catalyst particle cross sections, reporting the enrichment of Fe (*10*, *19*, *36*–*38*) and Ni (*12*, *18*, *36*, *39*) in a 1- to 5-μm-thick surface layer. All authors agreed that after initial deposition, both metals are either immobile or have very low mobility (*12*, *18*, *36*, *39*).

Ni contaminations in the feedstock are mainly associated with porphyrin or porphyrin-like complexes, that is, large molecules (*40*) that are decomposed soon after contacting the FCC catalyst particle (*12*) and before entering the smallest pores (*18*), Kugler and Leta (*12*) suggested that the large, Ni-carrying porphyrin complexes predominantly decompose at diffusion barriers, for example, in pores that are narrow or that have been narrowed through pore clogging caused by earlier metal deposition.

Two types of Fe are present in an FCC unit: (i) organic, finely dispersed Fe originating from the feed and/or hardware corrosion and (ii) inorganic, particulate Fe stemming from hardware or soil contamination (also called “tramp Fe”) (*36*). Tramp Fe particles are deposited on the surface of the FCC catalyst particle because they are too large to enter the particle through pores and can form highly localized nodules rich in Fe (*36*). The organic Fe (for example, naphthenates) is also deposited mainly in a surface layer of a few micrometers thickness because of its large molecular size (*38*). In combination with Na, another poisonous contaminant present in FCC, Fe can lower the melting point of silica to temperatures below those typically experienced in FCC units, leading to vitrification of the particle surface and the collapse of the surface pore structure. This effect leads to the observed formation of valleys and nodules in the particle surface (*36*).

On the basis of these earlier studies and the findings reported here, which are in excellent agreement with the proposed mechanisms, it can be concluded that the deposition of Fe and Ni from organic contaminations follows the same principle, leading to similar concentration profiles and pore blocking effects (Figs. 3 to 6): both metals are transported into the FCC catalyst particle by large molecules, which have a limited travel range in the pore network of the particle. When reaching a diffusion barrier, the large metal-carrying molecules are decomposed, depositing the metals in the pore channel. This further decreases the pore size, generating an even stronger diffusion barrier for subsequent molecules and ultimately leading to a self-enhancing process of pore clogging through metal deposition.

However, the data presented in this work also suggest differences in the deposition mechanisms for Fe and Ni. One difference was that Ni deposition has a stronger effect on pore narrowing than Fe, particularly at an early phase in the catalytic life of the particle (Fig. 4, middle). One possible explanation for this observation is a temporal fluctuation in Ni and Fe contamination levels in the feedstock, leading to stronger Ni deposition during a specific time period in the particle life. Another reason could be that Fe is mainly present as tramp Fe and therefore preferentially deposited at the surface and less so in the pores of the particle. Finally, if Ni-transporting molecules are preferentially smaller and/or can diffuse more easily into the particle than Fe complexes, more Ni than Fe will be deposited in pore channels. The latter case would explain why Ni (unlike Fe) continues to accumulate (presumably through meso- and micropores) even when many of the surface macropores are blocked (Tables 1 and 2). Another difference is that additional deposition of Fe occurs through particulate Fe oxides at the surface that can, together with the deposited organic Fe and accumulated Na, lower the melting point of the silica phase in the particle surface. For older FCC catalyst particles, that is, higher Fe concentrations, this can lead to surface vitrification and the collapse of surface pores, further restricting access to deeper parts of the particle. At this stage, metal-transporting molecules can hardly enter the particle, and further metal accumulation happens mainly through deposition at the surface. This effect can explain the observed shifts and the shoulder in the radial distribution of Ni (Fig. 3). That is, whereas Ni accumulation in the LML sample is dominated by porphyrin transport and deposition in the pore network, the beginning vitrification in the MML and HML samples starts to alter this deposition mechanism by changing the properties of the particle surface. Ni is no longer transported into the pore network of the particle but is deposited at the Fe-rich surface layer, causing a shoulder in the radial concentration plot in the HML sample, which indicates lower Ni concentrations at the surface than in slightly deeper parts of the HML sample. This mechanism explains the observed anticorrelation of Fe and Ni in terms of concentration and macropore clogging at later stages of the particle catalytic life. These observations further suggest a growing Fe-rich surface layer on older ECat particles, caused by the continuous deposition of Fe at the surface. This effect could ultimately change individual particle behavior in the many-particle ensemble, for example, through increased interparticle forces between spent ECat particles, as reported in the literature (*41*), that can lead to particle agglutination and hence decreased activity.

In summary, the findings reported in this work provide an unprecedented, detailed view on the changing internal macropore structure of aging FCC particles. We were able to correlate the 3D distribution of Ni and Fe to changes in porosity and pore connectivity in fresh and aged catalysts, in turn linking reduced catalytic activity to highly localized pore clogging. The results elucidate an important, as yet unknown aspect of metal poisoning: Fe and Ni accumulation is strongest early in cycle life and is concentrated at the particle surface and in a near-surface layer, whereas the inner core remains relatively clear of these metals, resembling the pore structure of a fresh particle. This inner, relatively unimpeded and interconnected pore network remains functional for reaction and is likely still accessible, although less efficiently, by meso- and micropores. Its presence explains the remaining functional activity of the catalyst, as confirmed by a small but quantifiable accessibility index and a measurable catalytic conversion rate, even for particles with the highest metal loading. Our results complement previous work that has shown that V (not studied in this work) can penetrate more deeply into the FCC particle, mainly affecting zeolite structures, rather than macropore radius, connectivity, and accessibility (*6*, *8*, *12*).

We expect that these new insights about catalyst aging could significantly influence future FCC catalyst and even reactor design, for example, by designing catalyst particles with an outer surface layer that is lean in zeolite material and rich in huge macropores. As a consequence, these very large macropores will allow for increased metal deposition without causing the currently observed access restriction to the internal pore space of the catalyst.

Finally, the developed analytical approach is valuable not only for investigating FCC particles but also for other catalyst systems, and can be applied even more broadly, for example, to geological studies of minerals relevant for CO_2_ sequestration, or for understanding changes in the pore network of particles in battery electrodes.

## MATERIALS AND METHODS

### Samples

We examined fresh FCC catalyst particles and ECat particles of varying catalytic age. The ECat particles were obtained from an industrial FCC unit, more specifically from the regenerator, so that most of the coke was already removed from the catalyst. Typically, a few tenths of a wt % of coke are still present on the ECat, which was removed through calcining the particles (see next section). To perform a proper evaluation of the changes in the FCC catalyst throughout its commercial life cycle, we used an ECat sample from a refinery that had been using a single catalyst vendor and a single FCC catalyst for a considerable amount of time. The FCC catalyst studied in this work was a standard residue cracking catalyst that did not contain any additives.

### Density separation of ECat particles

Before density separation, the ECat sample was sieved using a 45-μm sieve to isolate the particles with a diameter less than 45 μm (because of size restrictions presented by the penetration depth of x-rays and the depth of focus in TXM analysis). After sieving, the ECat sample containing only particles smaller than 45 μm was calcined at 600°C for 4 hours to remove any residual coke. The density separation was performed using a 50-ml titration burette, and density-separated batches of ECat particles were obtained using a method similar to a sink-float density separation method (*42*). In the sink-float method, a catalyst sample is added to a solvent mixture; the fraction with a density lower than that of the solvent mixture will float on the mixture, whereas the fraction with a density higher than that of the solvent mixture will sink. By adjusting the ratios of the light solvent acetone and heavy solvent diiodomethane (DIIM), the density of the mixture can be regulated. In our method, the density of the solvent mixture was adjusted until the solvent ratio was about 7:1 DIIM/acetone (v/v). Because this method separates particles on the basis of skeletal density, it can account for very large accessible voids within particles [as seen, for example, with SEM (*10*, *18*, *19*) or micro-CT (*21*)], whereas large inaccessible voids might lead to an incorrect classification in individual cases. However, the individual particles we imaged did not contain large voids, and hence, density was a reflection of metal deposition because of age. Arbitrarily selected single particles of each age group were then studied by SEM-EDX and TXM (see “Particle surface analysis” and “Full-field TXM”).

To achieve a separation for a larger quantity of catalyst required for activity testing and further characterization, a custom-made elongated separation funnel was used to achieve a columnar suspension similar to the burette used in the initial separation. These larger quantities of density-separated catalyst were then used to conduct a wider array of tests using bulk measurement techniques (that is, WDX analysis and catalytic performance testing; results are summarized in Table 1). The skeletal density of each density-separated batch was measured with a helium pycnometer, confirming the sample separation.

### Particle surface analysis

The different density groups, together with a sample of fresh catalyst, were then analyzed with respect to surface elemental composition using SEM-EDX and found to have a gradient in surface metal concentration directly correlated to their increasing age/density (Table 1). On the basis of this correlation between density/age and metal loading of Fe, Ni, and V, the density-separated batches were therefore labeled FRESH, LML, MML, and HML, indicating the fresh sample, low metal loading, medium to high metal loading, and high(est) metal loading, respectively.

### Bulk (multi-)particle analysis

Quantitative bulk elemental analysis was performed using a matrix calibrated WDXRF spectrometer, confirming the positive correlation of metal loading (Fe, Ni, V) with catalyst density/age (Table 1).

Micro- and mesopore surface area as well as micropore volume measurements were performed using nitrogen adsorption at 77 K, with 60 adsorption and 30 desorption points measured in the range of 0.000001 to 1 *P*/*P*_0_, and 30 desorption points measured from 1 to 0.2 *P*/*P*_0_.

In addition to pore size distribution, we also measured the change in mass transfer rates, or accessibility, supposedly caused by metals blocking pores. For that purpose, an in-house developed accessibility test [Albemarle accessibility index (AAI)] was used to measure the liquid-phase diffusion of large asphaltene molecules into the catalyst (*9*). In this test, no chemical reaction is occurring; it only determines the diffusion characteristics of the specific catalyst samples. A petroleum fraction containing asphaltenes is dissolved in a solvent and circulated through an ultraviolet-visible (UV-vis) spectrometer. At time zero, the catalyst is exposed to the solvent, and the relative concentration of UV-vis absorbing molecules in solution is tracked by the spectrometer over time. As the FCC catalyst takes up the asphaltene molecules, the asphaltene concentration in the solution drops. The test measures the rate of uptake of the asphaltene molecules and returns an index value, which is a relative measure of the initial slope of these curves (molecule concentration in the solution versus time) and can range from 0 to 30. For our samples, the equivalent fresh catalyst measured a 9.0 index value, whereas age-separated catalysts showed a decrease from 4.9 to 2.4 as a function of density and age. The AAI value therefore provided a direct measure of relative changes in accessibility of the catalyst for large asphaltenic molecules.

### Catalyst performance testing

Laboratory-scale performance testing was carried out on each of the density-separated ECat fractions, using the fluid bed simulation test (*43*). The catalysts were tested for their activity using VGO, at a cracking temperature of 538°C. The 430°F+ conversion of each catalyst was determined at four different catalyst-to-oil (CTO) ratios: 3, 4, 5, and 6. The 430°F+ conversion (wt %) is defined as the fraction of the feed that has been converted to products boiling below 430°F and coke. Alternatively, the 430°F+ conversion can be defined as (100 − bottoms − LCO)/100, where bottoms and LCO are the fractions of the liquid products that boil at a temperature above 430°F. The results of the performance testing show a clear correlation between catalyst deactivation/age (and therefore the metal levels of Ni and Fe in the sample) and catalytic activity: the older the catalyst, the lower the 430°F+ conversion of the catalyst material. A fresh catalyst will be very active (have a high conversion and produce a lot of coke and gas) and minimize bottoms and LCO, meaning that the conversion for the FRESH catalyst will be approaching 100% (especially for higher CTO ratios).

### Full-field TXM

Whole, single catalyst particles from each of the density-separated batches (LML, MML, and HML) and a FRESH catalyst were examined using TXM. TXM measurements were performed at wiggler beamline 6-2C at the Stanford Synchrotron Radiation Lightsource, SLAC National Accelerator Laboratory (*26*). The x-ray microscope is optimized for photon energies in the range of 5 to 14 keV, with spatial and energy resolution of 30 nm and Δ*E*/*E* = ~10^−5^ [Si(111) crystal], respectively.

In TXM, monochromatic x-rays are transmitted through the sample and then focused onto the detector plane (a scintillator screen) by means of a zone plate, which acts as a Fresnel lens for x-rays and an objective lens for the microscope. This generates a magnified transmission image of the sample on the scintillator screen, which is then recorded by an optical microscope equipped with a complementary metal oxide semiconductor camera. The microscope achieves a single flat FOV of about 15 × 15 μm^2^ or 30 × 30 μm^2^ (dependent on the energy and x-ray optics used). The FOV in TXM can be further extended with processing techniques (mosaic imaging), allowing for extended FOVs of up to 200 μm^2^ (*44*). Furthermore, owing to the large penetration power of hard x-ray radiation and the large depth of focus (>50 μm) when using hard x-rays, thick samples (tens of micrometers) can be imaged in 3D using tomography. When combined with tomography, it is possible to achieve full 3D mapping of the object of interest, including its internal structure (for example, the porosity distribution).

In TXM, the image contrast is a measure of the x-ray attenuation coefficient, which is a function of density, the elemental composition of the absorbing material, and the x-ray energy. Thus, element-specific imaging can be achieved by using the abrupt increase in x-ray absorption at element-specific x-ray energies. The image contrast achieved by subtracting 2D images or 3D volumes collected above and below the x-ray absorption edge of a specific element is therefore exclusively caused by the concentration of the element of interest, and, in this way, provides a direct measure of the (2D or 3D) elemental concentration distribution within the sample (Fig. 2C) (*33*).

For this work, data collection was performed in mosaic tomography mode. Whole catalyst particles were imaged with 180 2D projection images with one-angle steps with a dwell time of 1 s and repetition of 10 to 20 images at each mosaic projection (Fig. 2A). On each of those sets of 2D projection images, tomographic reconstruction was performed using an iterative algebraic reconstruction technique (i-ART) (*44*, *45*), resulting in a 3D reconstruction of the sample (Fig. 2B). Mosaic tomographic measurements were performed at the x-ray energies corresponding to below and above the Fe K-edge (7.100 and 7.132 keV, respectively) and below and above the Ni K-edge (8.325 and 8.350 keV, respectively). As schematically shown in Fig. 2, the final 3D representation of each inspected sample consists of these four tomographic data sets, providing information about sample morphology (tomography at 7100 eV), 3D Fe concentration distribution (differential absorption contrast between the data collected at 7100 and 7132 eV), and 3D Ni concentration distribution (differential absorption contrast between the data collected at 8325 and 8350 eV).

The 2D resolution of projection images was 32 × 32 nm^2^ (pixel size; the actual 2D resolution was below 30 nm), and this was downsampled to 64 × 64 × 64 nm^3^ voxels for the 3D data. The achieved 3D resolution was estimated by FRC (2σ criterion) to 314 nm (see the Supplementary Materials). Most of this study has been focused on determining global or regional, but not specific, individual changes to the pore network, and elemental distribution, and thus accurate determination of the resolution is not as crucial an aspect in our conclusions.

### TXM data analysis

Data preprocessing, including image alignment and mosaic tiling, as well as the tomographic reconstruction of the data sets, was performed using the TXM-Wizard software package (*44*) and the i-ART (*45*). Reconstructed data volumes were analyzed for single-particle metrics (table S1 and Supplementary Materials) and visualized using Avizo Fire software. Furthermore, skeletonization of the particle pore space was performed using this software to establish the pore network. Self-developed code written in the Matlab technical computing language was then used to evaluate the established pore networks, taking into account pore network changes caused by Fe and Ni. The radial evaluation quantifying metal concentrations and related porosity changes was also performed using self-developed code in Matlab. See the Supplementary Materials for more details.

## Supplementary Material

http://advances.sciencemag.org/cgi/content/full/1/3/e1400199/DC1

## SUPPLEMENTARY MATERIALS

Supplementary material for this article is available at http://advances.sciencemag.org/cgi/content/full/1/3/e1400199/DC1 Materials and Methods Fig. S1. Schematics of the topological representation of the pore network. Fig. S2. FRC for estimate of 3D resolution. Table S1. Single-particle metrics from TXM tomography data. Table S2. Basic parameters of the established macropore network. Movie S1. Fe and Ni distribution on and in a single MML catalyst particle. Movie S2. Visualizing the changes to the pore network with the presence of Fe and Ni in the pores. References (*46*, *47*)
