# Supplementary material for: Life and death of a single catalytic cracking particle
Source: Sci Adv. 2015 Apr 3;1(3):e1400199. doi: 10.1126/sciadv.1400199 (PMC4640619; doi:10.1126/sciadv.1400199)
Supplement: http://advances.sciencemag.org/cgi/content/full/1/3/e1400199/DC1 [file supp_1_3_e1400199__index.html]

Science Advances | Science Advances

## Supplementary Materials

**This PDF file includes:**

- Materials and Methods
- Fig. S1. Schematics of the topological representation of the pore network.
- Fig. S2. FRC for estimate of 3D resolution.
- Table S1. Single-particle metrics from TXM tomography data.
- Table S2. Basic parameters of the established macropore network.
- Legends for movies S1 and S2
- References (*46, 47*)

Download PDF

**Other Supplementary Material for this manuscript includes the following:**

- Movie S1 (.mov format). Fe and Ni distribution on and in a single MML catalyst particle.
- Movie S2 (.mov format). Visualizing the changes to the pore network with the presence of Fe and Ni in the pores.

**Files in this Data Supplement:**

- Adobe PDF - 1400199\_SM.pdf
