# Supplementary material for: Life and death of a single catalytic cracking particle
Source: Sci Adv. 2015 Apr 3;1(3):e1400199. doi: 10.1126/sciadv.1400199 (PMC4640619; doi:10.1126/sciadv.1400199)
Supplement: http://advances.sciencemag.org/cgi/content/full/1/3/e1400199/DC1 [file 1400199_SM.pdf]

Science Advances

www.advances.sciencemag.org/cgi/content/full/1/3/e1400199/DC1

Supplementary Materials for

Life and death of a single catalytic cracking particle

Florian Meirer, Sam Kalirai, Darius Morris, Santosh Soparawalla, Yijin Liu, Gerbrand Mesu, Joy C. Andrews, Bert M. Weckhuysen

Published 3 April 2015, *Sci. Adv.* **1**, e1400199 (2015)
DOI: 10.1126/sciadv.1400199

The PDF file includes:

Materials and Methods
Fig. S1. Schematics of the topological representation of the pore network.
Fig. S2. FRC for estimate of 3D resolution.
Table S1. Single-particle metrics from TXM tomography data.
Table S2. Basic parameters of the established macropore network.
Legends for movies S1 and S2
References (*46*, *47*)

Other Supplementary Material for this manuscript includes the following:
(available at www.advances.sciencemag.org/cgi/content/full/1/3/e1400199/DC1)

Movie S1 (.mov format). Fe and Ni distribution on and in a single MML catalyst particle.
Movie S2 (.mov format). Visualizing the changes to the pore network with the presence of Fe and Ni in the pores.

Supporting information - TXM Data Analysis

Single Particle Metrics

Calculated single particle metrics based on particle morphology are summarized in Table S1.

The (degree of) anisotropy for a particle is a measure of its 3D symmetry and an indicator for structural alignment along a specific direction. It was determined as 1 minus the ratio of the minimum over the maximum eigenvalue of the data cloud obtained by mean intercept length analysis. Mean intercept length analysis is performed with the binarized particle volume and determines the number of (filled) voxels that intersect with a set of oriented rays sent through the volume at different angles. The mean number of intersecting voxels as a function of angle forms the data cloud then used in eigenvalue analysis. Closer equality between the minimum and maximum eigenvalues leads to higher isotropy (degree of anisotropy becomes 0), which means that there is no privileged structural direction (e.g. for an isotropic sphere), while the degree of anisotropy becomes 1 for total anisotropy.

Further geometric descriptions of the particles were obtained through the eigenvalues of the covariance matrix built from the second order central moments (analogous to the moments of inertia used to generate the inertia matrix). The elongation of each particle was determined as the ratio of the medium and the largest eigenvalue of the covariance matrix, causing elongated objects to have values close to 0 (a perfect sphere has an elongation of 1).

The flatness of each particle was determined as the ratio of the smallest and the medium eigenvalue of the covariance matrix, which means that flat objects have values close to 0 (a perfect sphere has a flatness of 1). The values found for flatness and elongation of the particles already suggest that the particles are fairly spherical which is confirmed by the sphericity or roundness value determined as $(\pi^{1/3}(6V)^{2/3})/A$, where V indicates the total particle volume and A the corresponding surface area of the total particle volume. The sphericity value is a measure for how spherical an object is and is 1 for a perfect sphere.

Finally, the equivalent (spherical) diameter defined as the diameter of a sphere of identical (total particle) volume V was determined as $(6V/\pi)^{1/3}$.

The total particle volume and the corresponding surface area of the particles were determined directly from the reconstructed tomography data without considering pores. The total particle volume and the total particle surface area therefore correspond to the volume and surface area of a particle of identical shape without any pores.

Together with the known average density from bulk measurements (Table 1) it was then possible to calculate the mass of every particle, which reflects density and size differences between fresh and aged particles. The uncertainties for those metrics as provided in Table S1 represent the standard deviations determined from data collected below the Fe and below the Ni edge. Uncertainties were found to be smaller than 2% in all cases indicating excellent reproducibility of the tomography data recorded at those two different energies. For the FRESH sample no measurements were performed at the Ni X-ray absorption edge because no Ni was detected in this sample (Table 1).

Finally, macro-pore volumes and corresponding macro-pore surface areas were evaluated and have been normalized to particle mass, expressed in cm^3/g and m^2/g respectively, for easier comparison with the corresponding meso- and micro-pore data reported in Table 1. Here, standard deviations were not only calculated from the data collected at multiple energies but additionally considering differences between the two evaluation methods for pore space determination, which will be explained in the next section (i.e. also considering method-related

uncertainties). The same uncertainties are assumed for the pore volumes determined above the absorption edges (Fe and Ni) as indicated by v.s. (*vide supra*).

When comparing porosity data reported in Tables 1 and S1, we note that macro- and micro-pore volumes of the FRESH sample were found to be almost identical (~0.06 cm^3/g), while corresponding surface areas differ by two orders of magnitude, which is in agreement with an expected, very different surface area to volume ratio of macro- and micro-pores.

By using energy-resolved TXM data it is possible to determine macro-pore volumes with and without considering pore blocking by Ni and Fe. This is achieved by separately evaluating the pore volumes using data collected below and above the X-ray absorption edge of the corresponding metal. Therefore we expect all macro-pore volumes determined without considering the concentration distributions of Fe and Ni to be similar to the macro-pore volume determined for the FRESH sample. This is indeed confirmed, as shown by the normalized macro-pore volumes determined without considering pore blocking by Fe and Ni (Table S1, in % total particle volume (% TPV); also shown in the next row as macro-pore volume in cm^3/g). However, while the reported micro-pore volume clearly decreases with increasing metal loading (Table 1), such a clear trend is not observed for the macro-pore volumes considering the metals (Table S1, macro-pore volumes determined considering pore blocking by Fe and Ni), except for a drop in macro-pore volume between the FRESH and the other samples. This drop (0.046 in Fresh to below 0.04 in LML, MML and HML) is consistent with the drastic increase in metal loading seen from FRESH to LML. Moreover, it is striking that for all particles the changes in macro-pore volumes and macro-pore surface areas due to the presence of the metals Fe and Ni are not significant within combined uncertainty levels and similar to the change observed in the FRESH sample. This can be explained by the fact that much of the deposited Fe remains on the surface, i.e. as seen by SEM-EDX (Table 1) where surface Fe concentrations were reported to be larger than corresponding average bulk concentrations as reported by WDXRF. These observations suggest that Fe and Ni are highly localized, namely at the particle surface and in a near surface layer. Changes in the pore volume caused by those metals are therefore also highly localized and take place only in a small sub-volume of the aged particle (more specifically at the surface or in surface near regions, as confirmed by the plots reported in Fig. 3), while the main, inner particle volume remains largely unaffected. This large, inner volume, where metal concentrations are low and at the same level as in the pristine FRESH sample, dominate the evaluation of pore changes if performed at the total particle level (i.e. when inspecting the average change for whole, single particles).

Determination of Uncertainties

At this point it is necessary to explain some fundamentals of the approach developed for determining particle pore volumes and related uncertainties. The assessment of the uncertainty in the pore-space determination is based on using two different methods for assigning pore space, which employ different methods for noise correction. The nature of the two noise correction procedures causes Method 1 to generally underestimate pore space reduction by the metal while Method 2 always overestimates the reduction. In this way we established upper and lower limits (i.e. an error margin) for metal caused pore-space reduction and can use the average as a noise corrected value. As seen in Fig. 3 the influence of noise is (as expected) mainly affecting small changes caused by small metal concentrations, which are close to the detection limit of the method. Naturally the error margin becomes larger for a smaller inspected volume as then the influence of noise becomes more significant: in Fig. 3, when probing deeper into the particle, the volume of each particle shell at a specific distance from the surface becomes smaller with increasing distance from the surface, and thus the error margins increase. One limitation of the method is that we assume the presence of the metal and that it affects the pore space. As a consequence, the average pore volume reduction is overestimated if the metal is not present in

measurable amounts and does not cause a significant pore blocking. However, also in this case, although error margins are shifted together with the overestimated average value, the calculated uncertainty still provides a good estimate for the error margins. Based on this determination of the uncertainty in porosity changes caused by the metals, we conclude that porosity changes below 20%, as mainly observed for Fe in the FRESH sample and related to small metal concentrations in all samples (Fig. 3, B), have to be considered below the limit of detection of our method (when applied to these FCC samples) and are overestimating the actual change. With increasing metal concentrations, this over-estimation becomes smaller and a comparison of the two evaluation methods provides a good estimate of the actual porosity change and its related uncertainty.

We can now understand the result reported in Table S1, showing that the FRESH catalyst particle exhibits a total pore volume reduction for Fe in the same range as all other particles, although it is known to contain very little Fe. As explained in the previous paragraph, this is a direct consequence of the elemental concentrations being close to the detection limit of the instrument: the main volume of this particle contains no or little metal affecting the pore volume and therefore the total, average pore change is overestimated.

Radial evaluation of elemental concentration and porosity changes

Utilizing the fact that the particles were found to be sufficiently spherical (see Table S1), we assessed the relative elemental concentrations of Fe and Ni as a function of particle radius. Particle segmentation into concentric shells has been performed using a 3-step process. In the first step, we determined the total particle volume (reported in Table S1) by binarizing the recorded optical density (OD) data into empty and filled voxels (using threshold 1 (th1)). We then filled the pores inside the particle volume using algorithms based on geodesic dilation as provided by the Avizo® Fire software. Further data processing was performed in Matlab®. In the second step the shortest (Euclidean) distance of every voxel to the closest particle surface was calculated, providing the distance of every voxel to the outer surface of the particle. Based on these distances the center of the particle, i.e. the voxel with the largest distance to the surface, was identified. In the third step voxels with equal distances to the surface were pooled, forming a shells of single-voxel (i.e. 64 nm) thickness. Starting from the surface, each particle was therefore segmented into a series of concentric 64 nm thick shells, accounting for the irregular shape of each particle. The porosity of each shell was then determined as the ratio of void space (volume of voxels in the shell assigned to pore space) to total space (volume of all voxels in the shell). As explained above, relative elemental concentrations were determined by the differential absorption contrast between data collected below and above the absorption edge of the metal. By combining information about pore space with the 3D elemental distribution we were able to correlate changes in pore space with the presence or absence of the specific element of interest.

The results of this evaluation are displayed in Fig. 3. The two evaluation methods explained in the section above define the upper and lower limits of uncertainty of the fan plots. For high metal concentrations, the uncertainty and any overestimation of the pore reduction is very small, while both increase at lower elemental concentrations, thereby slightly overestimating porosity change related to the metal. As expected, the uncertainty also becomes larger for smaller inspected volumes (shell volumes are smaller closer to the particle center). The Fe concentration in the pristine (FRESH) particle matrix is below or close to the detection limit of the TXM instrument, causing the average to be close to zero in each shell. In deeper shells of the particle, the Ni elemental concentrations also approach this detection limit. It is important to note that zero or negative average elemental concentrations within a shell do not mean that there is no metal in the shell but indicate that the majority of voxels contain no Ni (and therefore a decreased OD value above the edge). This explains why these shells can still show porosity changes due to Ni or Fe in the FRESH sample. The porosity change due to Fe detected in the FRESH sample can be

explained by (naturally present) Fe rich zones in the particle filler/binder (clay): when calculating the macro-pore space without Fe, these Fe 'hot-spots' can then become classified as pore space. It is not possible to unambiguously discriminate such naturally present Fe enriched zones in the filler from Fe introduced by the feedstock; we therefore consider the Fe related porosity change in the FRESH sample as an offset and limit of detection for porosity changes caused by Fe deposition (together with the above explained overestimation of pore space reduction for small metal concentrations). Unlike Fe, Ni is not expected to be part of the initial particle matrix (as confirmed by the absence of Ni in the Fresh sample, see Table 1), but is introduced by the feedstock; the detected Ni should therefore be mainly found in the accessible pore volume of the particle. This explains why Ni is causing similar porosity changes as Fe, although Fe concentrations were found generally higher than those of Ni (in agreement with WDXRF and SEM-EDX data, see Table 1): all of the detected Ni is introduced by the feedstock and available for pore blocking, while some of the measured Fe is not deposited but part of the particle matrix and therefore not blocking macro-pores. Ni is also expected to deposit in the micro- and meso-pore space of the particle; however, as only macro-porosity can be analyzed by the presented method thus we expect (and observe) that not all of the detected Ni is clogging macro-pores.

Skeletonization and pore network evaluation

In this step, we developed an approach to quantify and compare accessibility of entire catalyst particles in order to understand catalyst failure due to accumulation of Fe and Ni, which can cause narrowing of macro-pore channels (Fig. 4, case b), and blocking of surface access sites to macro-pores (Fig. 4, case c). The accessibility model is based on the feedstock intrusion direction, pointing from the surface of the particle towards its center, in order to investigate pore space accessibility with respect to possible routes of any material (feedstock) entering the catalyst particle. To perform this task, every measured pore space (as schematically depicted in Fig. 4, top) was represented by its topological skeleton, i.e. a thinned version of the pore shape consisting of points and lines equidistant to the shape boundary. After skeletonization, the pore volume is completely described by this set of points, lines, and corresponding distances to pore boundaries, which we identify as the pore network in the following. Skeletonization of the segmented pore volumes was performed using the Avizo® XSkeleton Pack software, by calculating the distance of every pore voxel to its closest boundary and then removing voxel by voxel from the segmented object until only a string of connected voxels remained ('thinning'). These resulting lines were then translated into points, segments (connecting points), and nodes (points where more than two lines meet) as schematically depicted in Fig. S1, forming a topological representation of the pore-network. The volume represented by the pore network is determined by summing the volumes of all pore channels, each represented by a cylinder, which is determined by the distance between two segment points (cylinder height) and their average distance to the shape boundary (cylinder radius) (see Fig. S1).

The resulting pore network volume is an estimate of the actual pore volume due to the interpolation of the shape boundaries. Furthermore, very small isolated pore volumes (e.g. single empty voxels) are removed in the thinning step. As shown in Table S2, the sum of these effects results in a smaller represented macro-pore volume than the macro-pore volume reported in Table S1 with a difference of up to 60%. However, the established topological pore network is not used to determine the actual pore volume, but to study pore connectivity, which is represented correctly. The volume covered by the network is exclusively used to assess the importance of sub-networks within the pore network, which consists of a set of isolated sub-networks not connected to each other, but describing a set of interconnected points (pores), i.e. isolated pore volumes in the particles. Further statistical analysis of the calculated pore-networks was performed in Matlab®. As an example, the first four rows in Table S2 list some basic

parameters of the established pore-network describing the pore volume calculated using Method 2 and data collected below the Fe X-ray absorption edge (i.e. not considering any pore blocking effects). The remaining rows list three measures identified as key parameters to describe the pore network and pore blocking effects in single FCC particles. These parameters were calculated using data collected at the four different X-ray energies (below and above the absorption edges of Fe and Ni respectively), using Methods 1 and 2 to determine error margins for pore volumes below the absorption edges.

The first parameter, the average pore radius, was determined as the average radius of all cylinders representing the pore channels as displayed in Fig. S1. Note that the average pore diameters were found between 64 to 128 nm, i.e. 1 to 2 voxels, and that the pore channel diameter resolution limit is 64 nm (a single voxel). This means that the macro-pore volume is dominated by small macro-pores with sizes approaching the meso-pore range (i.e. pore sizes < 50 nm) *(23)*. The second parameter reports the pore volume covered by the largest sub-network of the total macro-pore network. In all particles and at all energies a single, very large sub-network dominates the pore network, containing more than 94% of the total pore volume covered. Thus, more than 94% of the macro-pore space in these particles is interconnected. Based on this finding we established a third parameter, which counts the nodes of this largest sub-network that are located within a single voxel (64 nm) thick layer of the particle surface, i.e. the points of this sub-network connecting its pore volume to the outside of the particle. These surface nodes therefore act as entry points (or 'surface access points') for feedstock molecules, allowing them access to more than 94% of the pore volume of the particle. Finally, the numbers reported in Table S2 highlight differences between the established individual pore networks. The topology of the pore network of each individual particle is as distinct as the particle itself, which is why we have to compare relative changes in the three established key parameters due to the presence of the metals Fe and Ni. These comparisons are shown in Table 2 and Fig. 4, and are reported in percent of the value determined below the respective X-ray absorption edge of each metal. Note that the latter distinction into two pairs of macro-pore networks (one pair for each metal) is necessary because the X-ray absorption coefficient, which is the recorded value in an X-ray transmission experiment, changes as a function of energy. For small differences in the energy of the incident X-rays, the difference in the absorption coefficient diminishes allowing a direct comparison of recorded pore volumes. (This is always true, except when measuring across the absorption edge of a specific element of interest, which is intentionally used in differential absorption contrast imaging (i.e. elemental imaging) as explained above). However, when the incident X-ray energy is significantly different, the determined macro-pore networks will be different (although certainly similar) as well, which prevents, for example, the combination of the macro-pore network established below the Fe X-ray absorption edge energy (7100 eV) with data collected above the Ni X-ray absorption edge (8350 eV). For this reason, we evaluated each pair of collected data sets (one set for Fe and Ni respectively) individually, inspecting macro-pore networks and related pore blocking by Ni and Fe separately. The results reported in Fig. 4 and Table 2 are in excellent agreement with the surface metal loading reported by SEM-EDX analysis (Table 1) where, for example, only a very small increase in Ni concentration was detected between the LML and MML samples and a slightly stronger increase between the MML and HML samples.

Finally we want to point out that due to the energy dependence of the X-ray absorption coefficient and also the influence of noise, the individual macro-pore networks established at the Fe and Ni X-ray absorption edges are similar, but should not be directly compared (as seen in the distribution of access points for Fe and Ni in Fig. 5 and Fig. 6). However, a comparison of the evaluated average morphological parameters (e.g. average pore radius, or covered pore volume), as well as the relative changes due to the presence of metals (pore radius change, change in

number of access points) is valid, as these attributes are representative for the whole macro-pore network.

Estimate of the achieved 3D resolution

A Fourier Ring correlation (FRC) was performed in order to estimate the 3D resolution at which the signal is significantly above the noise level for the 3D datasets *(46)*. The full set of projection images were split into to two datasets such that each includes a distinct set of projection images: the first dataset contained images recorded at odd projection angles, the second dataset projection images recorded at even projection angles. FRC was performed for 300 (arbitrarily selected) reconstructed slice pairs from these datasets as described by Nieuwenhuizen et al. *(47)*. The FRC curve displayed in Fig. S2 was calculated as the average FRC of all 300 slice pairs. The resolution was determined using the intersection point of the FRC curve with the 2-σ curve. Using this criterion, the resolution calculated was 314.03 nm (0.1019 reciprocal pixels of 32nm size).

Figures and Tables

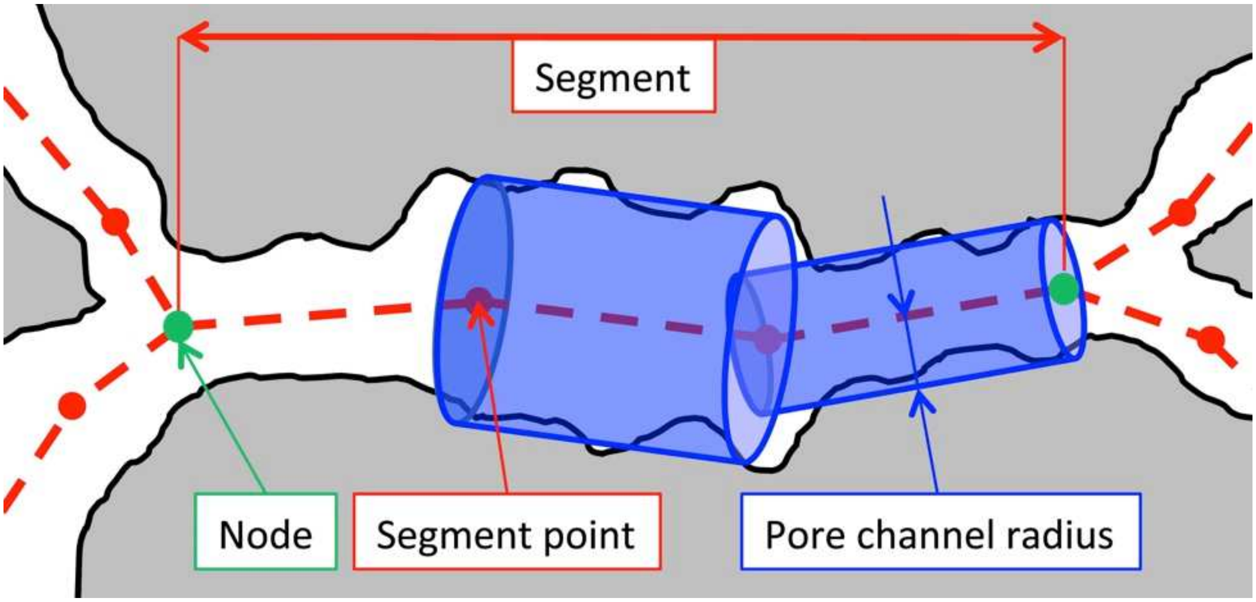

Fig. S1. Schematics of the topological representation of the pore network. The measured pore space is interpolated and fully described by a set of segment points, segments, nodes, and pore channel radii.

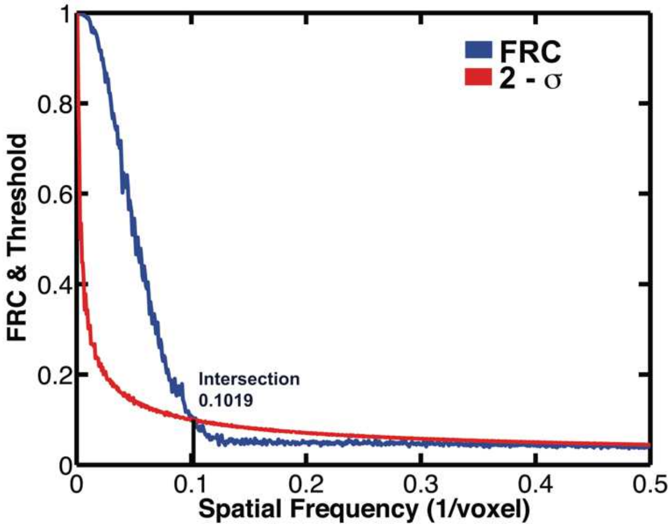

Fig. S2. FRC for estimate of 3D resolution. The achieved 3D resolution as estimated by the average FRC of 300 arbitrarily selected slices of the reconstructed 3D data using the 2-σ criterion. The intersection point at 0.1019 reciprocal pixels of 32 nm size suggests a 3D resolution of 314 nm.

Table S1. Single-particle metrics from TXM tomography data. For the FRESH sample no measurement was performed at the Ni edge because Ni was not detected in this sample (indicated by N/A (not available)). Standard deviations were calculated from data collected for the same particle, but at multiple energies. Standard deviations for macro-pore volumes and surface areas below the absorption edges were calculated with the two evaluation methods for pore space determination (i.e. considering method-related uncertainties). The same uncertainties were assumed for the pore volumes determined above the Fe and Ni absorption edges (indicated by vide supra, v.s.).

Metric	FRESH	LML	MML	HML
Anisotropy [1]	0.374	0.166	0.429	0.297
Elongation [1]	0.713	0.933	0.789	0.838
Flatness [1]	0.878	0.894	0.724	0.839
Roundness / Sphericity [1]	0.818	0.848	0.847	0.829
Equivalent Diameter [µm]	34.0	49.8	40.9	45.4
Total Particle Volume (TPV) [μm^3]	20650 ± N/A	64765 ± 92	35924 ± 36	48955 ± 54
Total Particle Surface Area [μm^2]	4449 ± N/A	9118 ± 68	6205 ± 7	7670 ± 139
Particle mass based on bulk density [ng]	58 ± N/A	191 ± 0.3	106 ± 0.1	145 ± 0.2
Macro-pore Volume [μm^3]	3289 ± 459	9189 ± 916	4703 ± 282	6793 ± 831
Macro-pore Volume, in % of TPV	15.9 ± 2.2	14.2 ± 1.4	13.1 ± 0.8	13.9 ± 1.7
Macro-pore Volume [cm^3/g]	0.057 ± 0.008	0.048 ± 0.005	0.044 ± 0.003	0.047 ± 0.006
Macro-pore Volume considering Fe [cm^3/g]	0.046 ± v.s.	0.039 ± v.s.	0.039 ± v.s.	0.035 ± v.s.
Macro-pore Volume considering Ni [cm^3/g]	N/A	0.036 ± v.s.	0.036 ± v.s.	0.039 ± v.s.
Macro-pore Surface Area [m^2/g]	1.342 ± 0.163	0.770 ± 0.142	0.618 ± 0.058	0.865 ± 0.120
Macro-pore Surface Area considering Fe [m^2/g]	1.172 ± v.s.	0.772 ± v.s.	0.645 ± v.s.	0.828 ± v.s.
Macro-pore Surface Area considering Ni [m^2/g]	N/A	0.603 ± v.s.	0.558 ± v.s.	0.771 ± v.s.

Table S2. Basic parameters of the established macropore network. Each network and its corresponding parameters represent a single particle pore volume. Uncertainty levels for pore volumes below the absorption edges were determined using two evaluation methods.

Pore volume	Metric	FRESH	LML	MML	HML
Below Fe absorption edge	*Number of Sub-networks [1]*	9832	30293	10976	24717
	Total Number of Segments [1]	569274	936022	386632	810803
	Total Length of All Segments [µm]	702218	1302662	610988	687969
	Total Volume Covered [μm^3]	2453	5673	4437	2780
	Average pore radius [nm]	32.9 ± 0.3	36.3 ± 1.6	40.4 ± 2.2	34.9 ± 1.0
	Volume Covered by Largest Sub-Network [%]	95.0 ± 4.0	95.2 ± 2.7	97.9 ± 1.0	94.2 ± 3.4
	Number of Surface Access Points of Largest Sub-Network [1]	7707 ± 872	9208 ± 1018	515 ± 149	10681 ± 1080
Above Fe absorption edge	*Average pore radius [nm]*	32.3	33.1	34.9	32.5
	Volume Covered by Largest Sub-Network [%]	97.9	97.1	98.9	94.5
	Number of Surface Access Points of Largest Sub-Network [1]	6297	5518	26	667
Below Ni absorption edge	*Average pore radius [nm]*	N/A	46.4 ± 6.2	46.3 ± 1.2	38.0 ± 1.5
	Volume Covered by Largest Sub-Network [%]	N/A	95.2 ± 1.7	97.9 ± 1.0	97.1 ± 2.5
	Number of Surface Access Points of Largest Sub-Network [1]	N/A	526 ± 86	126 ± 8	5447 ± 746
Above Ni absorption edge	*Average pore radius [nm]*	N/A	35.8	37.0	32.7
	Volume Covered by Largest Sub-Network [%]	N/A	95.2	97.3	98.1
	Number of Surface Access Points of Largest Sub-Network [1]	N/A	98	19	0

Movie S1. Fe and Ni distribution on and in a single MML catalyst particle. Movie showing the distribution of Fe (Red to White) and Ni (Blue to Green) on the MML particle. The slice through shows that there is a low concentration in the interior whereas the majority of high intensity metals is situated on or near the surface of the particle.

Movie S2. Visualizing the changes to the pore network with the presence of Fe and Ni in the pores. A flight through the HML particle following a path in the pore network from node (red spheres) to node. The nodes are connected by colored lines (segments) representing the pore channels and their color (ranging from blue to red) indicates the radius of the pore channel (see Fig. S1): blue indicates narrow tubes and red channels with large radius. After 48 seconds the measured Fe in the pores (red voxels) is added, showing how some pore network paths are now blocked by the metal (e.g. at second 52 in the movie). After second 59 the measured Ni in the pores (green voxels) is added as well, additionally blocking pore space.
